# PRIAMOS: A technique for mixing embedding media for freely adjusting pH value and refractive index (RI) for optical clearing (OC) of whole tissue samples

**DOI:** 10.1111/jmi.70022

**Published:** 2025-08-08

**Authors:** Ulrich Leischner, Martin Reifarth, Monika S. Brill, Florian Schmitt, Stephanie Hoeppener, David Unnersjö Jess, Hjalmar Brismar, Ulrich S. Schubert, Rainer Heintzmann

**Affiliations:** ^1^ Microscopy Department Leibniz Institute of Photonic Technology Jena Germany; ^2^ IZKF, Interdisziplinäres Zentrum für Klinische Forschung University Hospital Jena Jena Germany; ^3^ Nanobiophotonik, Institute of Physical Chemisty Institute of Physical Chemistry and Abbe Center of Photonics Friedrich Schiller University Jena Jena Germany; ^4^ Polymer Materials and Polymer Technologies Institute of Chemistry University Potsdam Potsdam Germany; ^5^ Fraunhofer Institute of Applied Polymer Research Potsdam Germany; ^6^ Institute of Neuronal Cell Biology Technical University of Munich Munich Germany; ^7^ Munich Cluster of Systems Neurology (SyNergy) Munich Germany; ^8^ Laboratory of Organic and Macromolecular Chemistry (IOMC) Friedrich Schiller University Jena Jena Germany; ^9^ Jena Center for Soft Matter (JCSM) Friedrich Schiller University Jena Jena Germany; ^10^ Science for Life Laboratory, Department of Applied Physics Royal Institute of Technology Solna Sweden; ^11^ Science for Life Laboratory, Department of Women´s and Children's Health, Karolinska Institutet Solna Sweden; ^12^ Helmholtz Institute for Polymers in Energy Applications Jena (HIPOLE Jena) Jena Germany

**Keywords:** high‐index fluids, Light‐Sheet Microscopy, Optical Clearing, Refractive Index Matching, Sample Embedding

## Abstract

Investigations of biological samples often require sample transparency, which is achieved by embedding the sample in a high‐refractive index (RI) medium to obtain a homogenous RI distribution in the sample, referred to as optical clearing (OC). Here, we introduce a method for designing embedding media with an increased RI by increasing molecular orbitals, which is achieved by replacing elements in molecules commonly used for OC with elements possessing a more pronounced polarisability. Briefly, we took the established embedding medium Glycerol and exchanged the OH‐groups by Thiol‐groups, resulting in an embedding medium with very similar properties, but with a higher refractive index. We describe a procedure—abbreviated PRIAMOS—to render biological samples transparent using an RI‐matching liquid, which we refer to as **p**H‐value and **R**efractive **I**ndex **A**djustment by **M**ixing highly polarisable molecular **O**rbital **S**ubstances. We focus on optical clearing in three‐dimensional tissue samples whilst preserving fluorescence of fluorescent labels. The clearing procedure requires 2–3 days, yielding highly transparent samples, preserving the fluorescence of fluorescent proteins like the yellow fluorescent protein (YFP). This is demonstrated on mouse brain samples, imaged with standard confocal microscopy down to 1.6 mm depth, as well as on kidney samples. Mixtures of monothioglycerol, dithioglycerol and tributylamine achieve RI values between 1.52 and 1.57, and an acidity equivalent to pH values between 5 and 8. Our PRIAMOS approach can serve as a guideline for optimising optical clearing protocols.

## 1 INTRODUCTION

Biological samples are usually opaque, since propagating photons are strongly scattered on their way through the sample. This photon loss prevents light microscopy to map the 3D‐arrangement of proteins and the shape of cells in deep layers of an uncut, whole‐mount sample. There are several chemical procedures described for making the sample transparent,^1^, ^2^, ^3^, ^4^, ^5^, ^6^, ^7^, ^8^, ^9^, ^10^, ^11^, ^12^, ^13^ minimising scattering loss, thus, enabling the observation of deep layers inside the sample by optical microscopy. With such optical clearing the 3D‐arrangement of proteins and cells throughout the depth of an isolated organ was reported.^1^, ^5^, ^6^, ^7^, ^8^, ^9^, ^11^, ^12^, ^13^ Most of these procedures are based on RI matching, which is achieved by adding the embedding media to the sample. Embedding with such a medium replaces the hydrophilic and water‐based components of cells, which are found in the cytosol, extra‐cellular space, etc., by a medium with an RI similar to proteins, DNA or lipids. The biological specimens have to be dehydrated using solvents, such as ethanol, or tetrahydrofurane (THF), enabling an immersion of the sample in a hydrophobic organic solvent environment. This replacement procedure minimises micro‐scale refractive index inhomogeneities, enabling a straight propagation of light through the tissue, allowing for the acquisition of images in deep tissue layers.

Spalteholz,^5^ an early pioneer of optical clearing, used a mixture of benzyl alcohol and benzyl benzoate (abbreviated BABB). This initial protocol is still used^14^, ^15^, ^16^, ^17^, ^18^ and provides good results in terms of sample transparency, but it displays the disadvantage that fluorescent proteins (FPs) like GFP or YFP quickly reduce their fluorescence brightness, unless special care is taken to avoid such loss.^16^, ^19^

Since many researchers utilise transgenic fluorescent tags to label certain types of cells, the preservation of protein fluorescence is crucial for cell detection and identification, rendering BABB‐based approaches rather impractical. Other clearing protocols preserve the aqueous environment of the embedding medium, and increase its refractive index by dissolving molecules with a higher refractive index, for example, urea,^9^ sugars,^3^, ^7^, ^20^, ^21^, ^22^, ^23^ other water‐soluble molecules^8^, ^24^, ^25^, ^26^, ^27^ or solvents.^27^ However, these water‐soluble substances do not achieve the high refractive index of BABB of *n* = 1.56, an optimum refractive index for best transparency as already found in the early study.^5^ Additionally, the highly concentrated solutions suffer from high viscosities, akin to honey, which leads to very long durations of the diffusion‐based clearing process with time spans of sometimes 1 to 2 months.^9^ As an additional drawback, small air bubbles that can evolve during the embedding procedure are trapped in the highly viscous solutions, which can be problematic if they are located in the imaging pathway of the microscope chamber. A final problem arises during the imaging step: even though several embedding substances can be screened for an optimised OC performance, objective lenses are often optimised for samples possessing a particular RI, and sample embedding in exactly these conditions is crucial in order to obtain good images. Common commercial objective lenses are optimised for a few RI values, such as water, for oil‐immersion, for glycerol‐immersion, and for the embedding substance BABB.^28^, ^29^, ^30^, ^31^ Tailored objective lenses exhibit a design with a correction collar for correcting the refractive index of the embedding medium up to 1.52. However, given the fact that good RI matching results were obtained using substances with an RI ≥ 1.52,^5^, ^14^, ^15^, ^16^, ^17^, ^18^ it would be highly useful to identify embedding media with RI in that range, ideally values matching the refractive index of BABB or immersion oil, as we could then use existing objective lens systems for image acquisition (e.g., Leica BABB objective, HCX Apo L20× /0.95 IMM).

It is therefore our aim in the present study to identify substances with an RI similar to BABB for OC, which enables us to use a BABB‐optimised optical system. The substance is, furthermore, supposed to possess a low viscosity to ensure rapid sample diffusion, ideally allowing OC in a time span of a few days. The RI matching procedure should be preserving the fluorescent of FPs. Since aromatic solvents are known to quench the fluorescence of GFP,^22^ we avoided such molecules. Ideally, the procedure should rely on water‐miscible solvents, rendering potentially invasive dehydration steps obsolete. Recently, Ou et al. could show, that molecules strongly absorbing in the visible region efficiently raise the dispersive part of the refractive index near the absorption band, facilitating optical clearing at wavelengths > 600 nm even under conditions compatible with live cell imaging.^32^ However, this process is incompatible with fluorescent labels showing absorptive and fluorescent characteristics at wavelengths below the aforementioned spectral range, such as, many common fluorescent labels, which is particularly problematic if multicolour imaging is aimed at.

To tackle these issues we propose using a mixture of monothioglycerol and dithioglycerol as embedding medium, along with the substance tributylamine for adjusting the acidity to pH values equivalent of 7 to 8, at which the fluorescent proteins GFP and YFP are functional.^33^, ^34^ We identified these substances applying a principle which we named PRIAMOS: **p**H‐value and **R**efractive **I**ndex **A**djustment by **M**ixing highly polarisable molecular **O**rbital **S**ubstances, which aims at optical clearing whilst preserving the fluorescence of FPs in three‐dimensional tissue samples. Our rationale was to replace commonly used OC agents with chemical analogues, in which one atom is substituted by a heavier atom. This modification adds atoms to the molecule, which are characterised by larger, thus, more easily polarisable molecular orbitals. As an enhanced polarisability of an electronic shell goes along with a higher RI, we were able to achieve high refractive index embedding conditions, which we demonstrate to be well suited for OC. Our data show that this educated search leads to compatible embedding media, which also perform well in sample clearing, preserving the fluorescence of proteins. We could show that our protocol is suitable for optical clearing of entire organs, whilst being well compatible with high‐resolution imaging.

## 2 MATERIALS AND METHODS

Tetrahydrofurane (THF) was purchased from VWR (CAS: 109‐99‐9, >99%, stabilised). Phosphate buffered saline (PBS) 1× was obtained from VWR (normal concentration of phosphate buffered isotonic salt solution, Dulbecco's formulation 1× sterile, VWR). Triethylamine was purchased from VWR (CAS: 121‐44‐8, for synthesis). Tributylamine (CAS 102‐82‐9, for synthesis, VWR) and dithioglycerol (CAS: 59‐52‐9) were purchased from Sigma Aldrich (≥98%) and TCI (≥95%). Monothioglycerol (CAS: 96‐27‐5) was purchased from Sigma Aldrich (≥99%) and TCI (≥98%). For immunostaining, (sc‐192) WT1 Antibodies (C‐19) (polyclonal rabbit antibody), supplied by Santa Cruz Biotechnology, against Wilms tumour protein (Human), were used as primary antibodies. Goat anti‐Rabbit IgG (H+L) Cross‐Adsorbed Secondary Antibodies, supplied by ThermoFisher Scientific, labelled with Alexa Fluor™ 488, were utilised as secondary antibodies.

### 2.1 Refractive index measurements of the embedding media

For RI determination, we prepared several mixtures of mono‐ and dithioglycerol (0, 20, 40, 60, 80, 100 vol.‐% v/v dithioglycerol in monoglycerol). Using an Anton Paar Abbemat 200 refractometer, we detected the RI using a 589.6 nm light source at a temperature of 20.00°C (n_D_).^20^ Each measurement was conducted as a triplicate.

### 2.2 pH value determination of the embedding media

In order to assess the acidic properties, we measured the pH values of monothioglycerol and dithioglycerol in a 0.5% solution of the compounds in water. For this purpose, we pipetted 500 µL of monothioglycerol in 100 mL dist., degassed water and detected a pH value of 6.2. Pipetting 500 µL dithioglycerol in 100 mL water resulted in a pH value of even 5.5. We thus repeated this experiment and adjusted all the pH values of all involved liquids to the range of 7 to 8 using a phosphate buffer in the first dehydration steps (50% and 70% v/v THF in water), and the base trietylamine for higher concentrations, with tributylamine for pure thioglycerols (using the pH‐sensor electrode InLab Science PRO from Mettler‐Toledo for organic solvents for pH determinations.

### 2.3 Mouse lines, husbandry, and brain sample preparation

All animal experiments conform to the German federal law. The protocols were examined by the Government of Upper Bavaria and approved by the ethics committee according to §15 German Animal Welfare Act, in agreement with the government of Upper Bavaria. The animals were housed in groups in individually ventilated cages (IVC) on dust‐free wood chips with red plastic houses, paper rolls, gnawing sticks, wood wool, and fed with standard mouse chow.

Mice were euthanised at 3 months of age. Thy1‐YFP mice (Jackson, stock number 3709) were transcardially perfused with a pre‐flush of PBS and 50 mL of 4 % PFA in PBS. Brains were dissected and post‐fixed in 4 % PFA in PBS overnight. We dehydrated the mouse brain sample using a rising concentration series of THF. The 50% THF was made using 1× PBS, the 80% and 90% THF solutions using 0.5× PBS (half‐concentrated solution as THF could not dissolve much salt), and we added 1% triethylamine to the pure THF to make it basic. The sample was initially immersed in the 50% THF solution, and we exchanged the embedding medium to the more concentrated medium after 1 h/mm sample diameter. For larger samples, for example, whole brains, this procedure takes about 1 day. We kept the sample in the last liquid (pure THF with triethylamine) overnight. We finally immersed the sample with a mixture of dithioglycerol (94.3% weight, 10 g) and tributylamine (5.4% weight, 0.57 g) and kept it again for 1 h per mm diameter of the sample. The diluted mixture of dithioglycerol and tributylamine features a pH value of 7.1 (500 µL in 100 mL water). The mixed embedding medium has a refractive index of 1.563 (at 550 nm wavelength) which well matches the Leica BABB objective lens (Leica HCX Apo L20× 0.95 IMM). For imaging, we used a confocal microscope from the Leica Imaging Center in Mannheim, equipped with the upper mentioned objective lens.

The sample was placed in a small petri dish filled with the immersion medium and moved around to find a promising position for a good acquisition and a non‐disturbed imaging of the whole sample, as well as to acquire good images also in deep layers. Voxel‐size: 0.11 × 0.11 × 0.8 µm. Objective: Leica HCX Apo L20 × 0.95 IMM. Beam‐splitter filter DD448/514,

We selected the hybrid detector for light detection, and the excitation beam splitter DD‐488/552. The pinhole size was set to 1 Airy unit. We acquired confocal images of the samples embedded in our medium using the BABB objective at a Leica confocal microscope at an excitation wavelength of 520 nm (4% laser intensity). Note, that already at a laser power of 5% intensity, the detector went into saturation.

The deconvolution was performed using a bespoke Matlab program (available upon request for evaluation purposes from R.H.), which tiled the data and submitted it to a Maximum Likelihood deconvolution routine running on a NVidia Gaming GPU. The optimisation used an L‐BFGS algorithm minimising a Poisson‐based likelihood functional for a fixed number of iterations per tile.

For the assessment of the mouse brain transparency, we took a mouse brain, cut it into two symmetrical hemispheres, and dehydrated them using increasing ratios of a THF/water mixture, with the same ratios as detailed in the prior paragraph. Deviating from that, triethylamin was used in all steps as opposed to the use of tributylamin in the final step. The volume‐ and weight ratios were kept identical to the previously described procedure, and the incubation time in each medium was 24 h. Subsequently, one hemisphere was submerged in dithioglycerol (containing 5.4 % weight of triethylamine), while the other one was kept in the 90% THF/water mixture. For comparison, both samples were removed from their media, placed in a petri dish on a millimetre paper and photographed after each incubation period.

### 2.4 Kidney sample preparation

We also applied PRIAMOS on kidney samples cleared with the CLARITY‐protocol and stained using immunochemistry. Kidney samples were cleared according to Unnersjö‐Jess^35^ and stained for WT‐1 (Wilms’ tumour 1) using a rabbit polyclonal primary antibody (Santa Cruz, sc‐192, 1:200, 2 days incubation) and a goat anti‐Rabbit Alexa488‐conjugated secondary antibody (1:200, 2 days incubation).

Wild‐type rats from Charles River, Germany, were used in all experiments. Animals were anaesthetised (intraperitoneal injection of pentobarbital), the aorta cut, and kidneys dissected. All experiments were performed in accordance with animal welfare guidelines set forth by Karolinska Institute and were approved by Stockholm North Ethical Evaluation Board for Animal Research.

Briefly, kidneys were dissected and immediately incubated at 4°C in hydrogel solution (1–2% v/v acrylamide, 0.0125%–0.025% v/v bisacrylamide, 0.25% w/v VA‐044 initiator, 4% PFA, 1 × phosphate‐buffered saline, PBS) over night. For gel formation, the sample was heated to 37°C for 3 h, trying to avoid oxygen by filling the sample tubes entirely with the polymerisation solution. Samples were removed from the hydrogel solution and immersed in clearing solution (200 mmol/L boric acid, 4% SDS, pH 8.5) and incubated for 1 day at 50°C. The kidney samples were next cut in 1.5 mm thick slices using a Vibratome (myNeurolab, St Louis, MO) and sections were afterwards immersed in clearing solution for 3 days at 50°C. Before immunolabelling, samples were incubated in PBST (phosphate buffered saline with Triton‐X, 0.1% Triton‐X in 1 × PBS) for 3 h at room temperature.

For immunostaining, (sc‐192) WT1 Antibodies (C‐19) (polyclonal rabbit antibodysupplied), supplied by Santa Cruz Biotechnology, against Wilms tumour protein (Human), were used as primary antibodies. As secondary antibodies, goat anti‐Rabbit IgG (H+L) Cross‐Adsorbed Secondary Antibodies, supplied by ThermoFisher Scientific, labelled with Alexa Fluor™ 488, were utilised. For all further immunolabelling steps, PBST was used as dilutant. Samples were incubated in a solution of the primary antibody for 24 h at 37°C and then washed in PBST for 8 h at 37°C, followed by an incubation with the secondary antibody incubation for 24 h at 37°C and washed for 8 h at 37°C.

For image acquisition, the sample was attached to a glass needle (a glass patch pipette, normally used in electro‐physiology for patching of nerve cells). The glass needle was attached to a metal block (similar to a candle to a candle‐holder), and we immersed this assembly group into a container filled with the immersion medium. This setup allowed to move the sample to a suitable position for imaging, as well as a levitating position somewhere in the middle of the container, to enable light‐sheets with large illumination apertures.

We acquired the images with a custom‐built light sheet microscope, which required an embedding medium with a refractive index of *n* = 1.52, which is equal to pure monothioglycerol. Accordingly, we transferred the CLARITY‐treated sample from the aqueous storage medium to the high‐RI medium thioglycerol by simply dropping it into pure monothioglycerol, and gained good results after some time (hours to 1 day as described in the experimental section) to allow for the diffusion of monothioglycerol into the sample. An adjustment of the pH value was not necessary in this case, as the samples were labelled with Alexa488‐conjugated antibodies, which fluoresce over a large range of pH values. A detailed analysis of the transparency at a larger depth was not possible due to the working distance of the objective being limited to 1 mm. However, the data shows that monothioglycerol with a refractive index of close to immersion oil (*n* = 1.52) can also yield high‐quality deep tissue imaging. For imaging, we used an air‐objective optimised for imaging through a 1 mm layer of (BK 7) glass (*n* = 1.52, Zeiss, LD APlan 10×/0.25) on which we mounted a ‘diving cap’ featuring an air gap and a 170 µm coverslip. A detailed description of the optical design can be found in Ref. (36). We used a configuration of light‐sheet‐imaging with a lateral sweep of the illumination line as described in Ref. (36), acquiring 14 images per slice, and combining the sharp illumination position to one image with good overall sharpness. We used a configuration with cubic voxels with a size of 600 nm in all directions. We used the Zeiss 10× LD A‐Plan 10×/0.25 (421240‐9900‐000) with 0.63× demagnification lens behind the tube lens, 488 nm as excitation wavelength and an emission filter (500–550 nm), 120 ms integration time per image, and a slit diameter of 7 mm, and the cylindrical focusing lens C30‐26HPX from Asphericon, resulting in a theoretical light sheet thickness of below 2 µm (without considering improper alignment and other tolerancing degradations in the optical setup).

## 3 RESULTS

Existing OC techniques can be grouped in three types of protocols, each with specific drawbacks: The first group uses organic solvents containing aromatic compounds as embedding medium.^5^, ^15^, ^16^, ^18^, ^37^ These protocols rely on an initial, rather invasive dehydration procedure as a first step. Even though the aromatic compounds diffuse rapidly into the tissue and render the sample highly transparent, protein fluorescence is quenched in the organic solvent. The second‐class dissolves hydrophilic molecules in water in order to modify the refractive index of the aqueous solution and use this highly concentrated solutions for sample immersion. Though these procedures do not require sample dehydration, they are often slow to fully immerse the sample due to the high viscosity of the resulting solutions.^7^, ^8^, ^11^ In addition, these solutions cannot reach refractive indices above 1.48, which is quite far from the optimum of 1.56 as found in the early publications of Spalteholz.^5^ Spalteholz et al. used saturated solutions to maximise the refractive index,^5^ and it can be expected that even higher refractive indices would result in even better transparency, which can, however, not be achieved with these techniques. The last class of OC techniques was initiated by the CLARITY‐protocol, in which the sample proteins are immobilised in a polymer matrix in a first step, followed by a removal of lipids using detergents, such as sodium dodecyl sulphate (SDS). As lipid structures provide the highest contribution of RI inhomogeneities, the lack of lipids renders the sample transparent to some extent, even without embedding it in a high RI matching medium. As a disadvantage, the CLARITY protocol is not easy to implement, and the sample preparation takes several weeks, requiring experimental planning long in advance.

**FIGURE 1:**
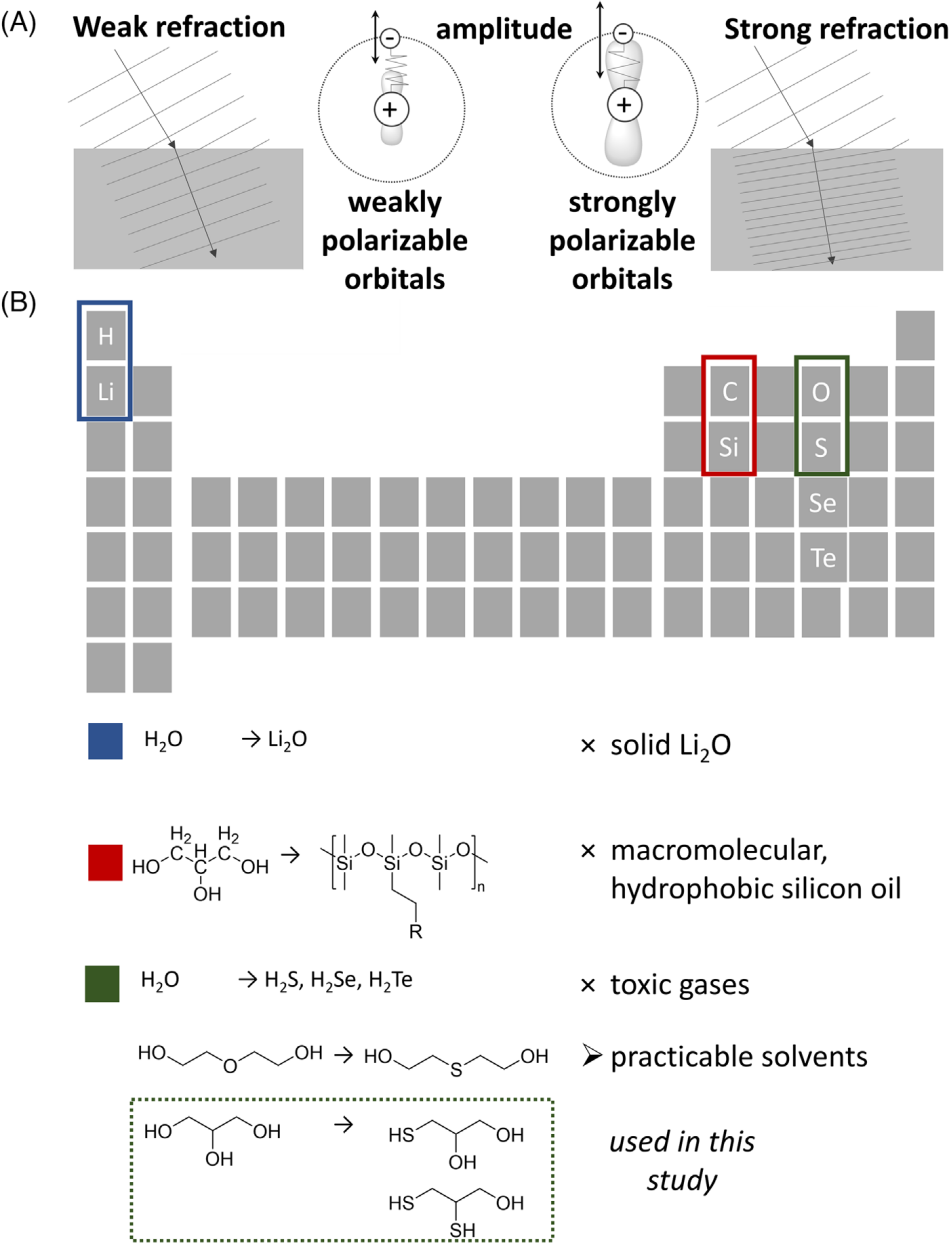
Rationale of PRIAMOS. (A) Schematic representation of the refractive index of a substance. Weakly refracting substances are characterised by weakly polarisable electron shells, whereas highly refracting substances exhibit well polarisable electron shells. The Lorentz oscillator model provides a simplified model of light refraction: Negatively charged electrons are bound to a nucleus possessing a positive charge via a spring, where badly polarisable orbitals correspond to a hard spring and easily polarisable electron shells are bound via a soft spring. (B) The PRIAMOS principle relies on the replacement of common clearing agents by clearing agents possessing an enhanced RI. On the way towards their identification, common structures were used, and atoms therein were replaced by atoms possessing larger orbitals.

We searched for an embedding medium that is better suited by adapting a more general view on the refractive index property of chemical substances as illustrated in Figure 1. Theory allows us to derive the refractive properties of a substance from the structural properties. The refractive index of a transparent, that is, non‐absorbing, substance reflects the ability of a molecule or an atom of being transiently polarisable. Light waves can be considered as an oscillation of the electromagnetic field, and when light propagates through the atom or molecule, the oscillating field causes the electrons of the atom or molecule to oscillate (Figure 1A).^39^ According to Maxwell's equations, these oscillating electrons reemit an electromagnetic wave with a different phase, and the superposition of the initial and reemitted wave cause a phase change per propagated distance in the medium (Figure 1A). This constant phase change with every passing layer is summarised as a shorter effective wavelength of the light *λ*_medium_ = *λ*_vacuum_/*n* in the medium, and the delay is characterised by the refractive index *n*.

In a simple picture, electrons that are more loosely bound in an atomic ensemble can oscillate more freely, with resonances (absorption) closer to the visible light and, therefore, cause an emission with more pronounced phase shift, resulting in a higher refractive index. These electron oscillations are particularly efficient for heavy atoms in which the electrons are located in larger atomic orbitals (and which also exhibit a low oxidation state, Figure 1A). Also, molecules with loosely bound, delocalised electrons tend to go along with a large refractive index (e.g., benzene contributes a high RI increment^40^). In contrast, molecules that exhibit more tightly bound electrons, for example, methanol, go along with a low RI. Although the above is a very simplified explanation of the refractive index and a better description would include the quantum‐mechanical theory, such a picture helps to identify suitable substances for RI matching. Another important property of a material to cause a high refractive index is its density: the denser atoms and electrons are present in a material, the stronger is their contribution per propagated distance, and therefore materials with a higher density exhibit also a higher refractive index.^40^

We tried to identify substances with higher refractive index by simply applying substances at which some atoms from a known molecule are substituted by atoms from the same chemical group of the periodic table with a larger electron orbital, and then checked if the substance is a suitable embedding medium (Figure 1B). Accordingly, we considered to exchange the hydrogen atoms in the embedding medium water (H_2_O) by lithium. This results in lithiumoxide (Li_2_O), which in fact does have a higher refractive index, but it is a solid and not a liquid, and therefore not suitable. We could also exchange the oxygen from H_2_O by sulphur, resulting in hydrogensulphide (H_2_S), but this is gaseous and not dense enough to exhibit a high refractive index. Hydrogenselenide (H_2_Se) or ‐telluride (H_2_Te) would possess a higher refractive index but are also gaseous and too toxic for practical use (Figure 1B).

We went on to other substances. Another well‐established substance for embedding biological tissue is glycerol. We can again try to find a similar substance by replacing single atoms from the same group, for example, the carbon with silicon. Such substances are referred to as silanols. Silicic acids tend to polymerise, forming silicates, which is a class of inorganic solid‐state materials, thus, non‐suited for our purposes. Organo‐silicon compounds, such as the well‐known silicon oils, are a manifold material class that can be equipped with a wide range of properties, ranging from hydrophobic to more hydrophilic substances. However, their macromolecular nature renders them viscous liquids, which impedes diffusion of the liquid into the material at a suitable timescale (Figure 1B).

Thiol‐variations of hydroxyl‐groups, where an SH‐group replaces an OH‐group in one or more positions, as well as thioethers ‐S‐ replacing ethers ‐O‐, are more promising. These compounds are stable, they are colourless liquids and exhibit a low viscosity. Finally, they are easy to purchase in a reasonable price range.

Glycerol is of RI of *n *= 1.47, whereas monothioglycerol of *n *= 1.52^41^ and dithioglycerol of a RI of 1.574,^42^ as given in the vendors datasheets or an internet database. Trithioglycerol has a refractive index of 1.571^43^ and therefore might not be better than dithioglycerol, which can be rationalised by a lower mass density than dithioglycerol. Mixing, thus, mono‐ and dithioglycerol, RI values in between 1.52 and 1.574 can be obtained, covering the refractive index of 1.52 matching an oil, and 1.56 matching the BABB objective, which we decided to use in the framework of this study (chemical structures are displayed in Figure 1B).^5^ Note that with our rationale, also other thiol compounds can be identified. As an example, thio‐sugars, where one hydroxy function is replaced by a thiol functionality, could serve as a potent OC agent. However, these are expensive and would therefore not be suited. Other examples comprise thioethers, which can be considered derivatives of ethers, whose oxygen is replaced by a sulphur atom. A prominent example is 2,2’‐thiodiethanol (TDE) as a sulphur analogue of diethylene glycol, whose utilisation as an OC agent has already been described in an earlier publication.^11^

**FIGURE 2:**
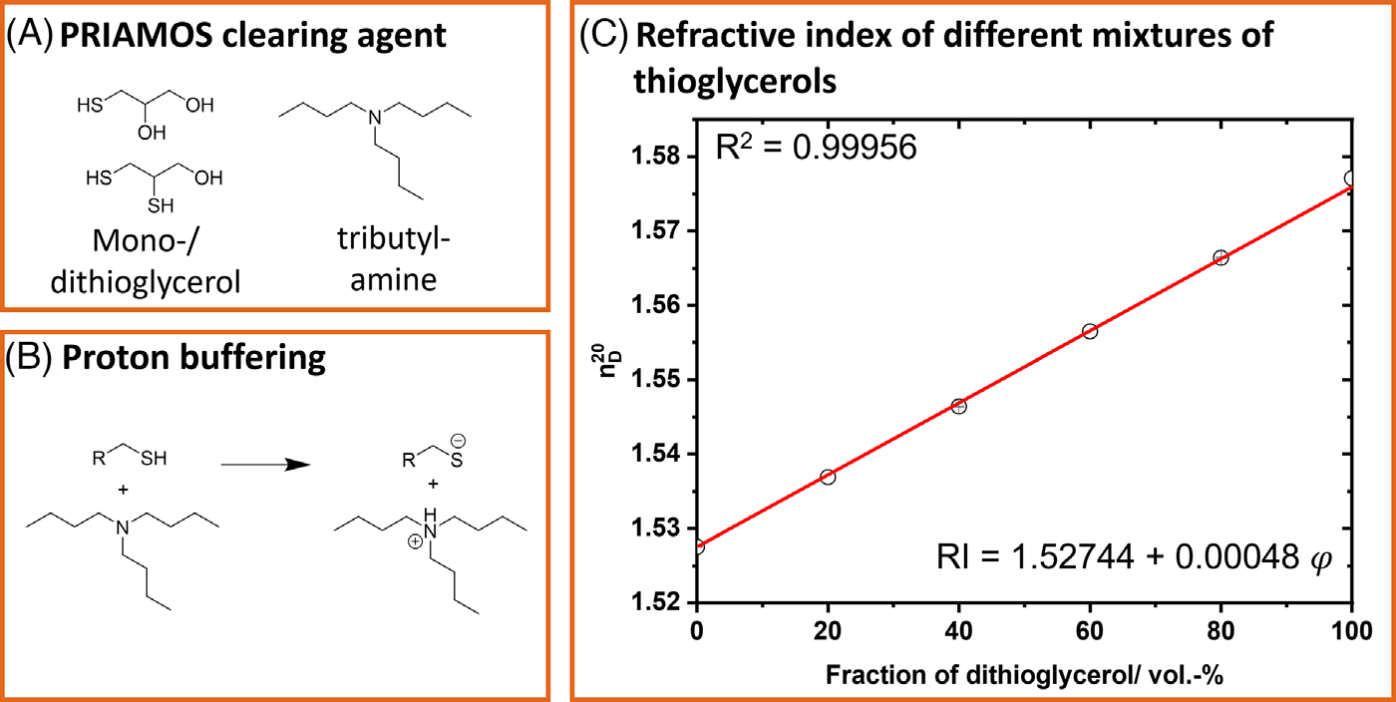
PRIAMOS procedure. (A) Chemical composition of the immersion medium. A mixture of mono‐ and dithioglycerol is used for optical clearing. (B) Tributylamine is used to buffer the thiol protons, which would otherwise interfere with the protein's fluorescence. (C) Refractive indices of binary mixtures of monothiol‐ and dithioglycerols. Each measurement was conducted as a triplicate.

A first practical check with monothioglycerol and dithioglycerol (chemical structures are depicted in Figure 2A) shows that both compounds are arbitrarily miscible with each other. Figure 2C, illustrating the overall RI of different binary mixtures of the thioglycerols, reveals a linear relationship between the fraction of dithioglycerol in the mixture and the RI value.

**FIGURE 3:**
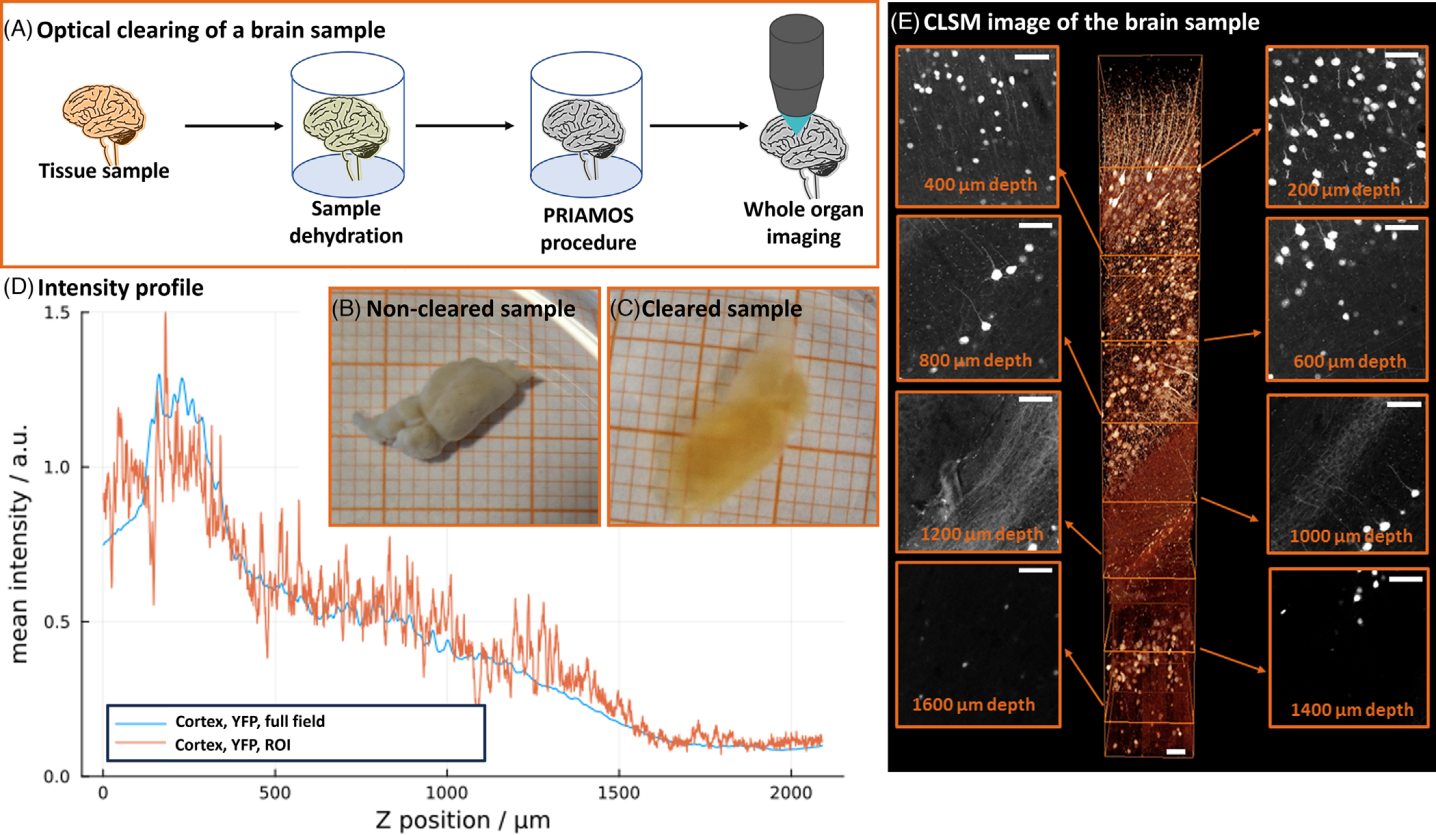
Whole mouse brain cleared with the thio‐glycerols. (A) Overall procedure of the clearing of the brain tissue sample. The tissue sample is first dehydrated using THF as a water‐miscible solvent, allowing for the immersion of the sample in the hydrophobic mixture of mono‐ and dithioglycerol. After clearing, the organ can be imaged using an ordinary confocal laser scanning microscope. (B) Non‐cleared sample in THF as a negative control and (C) sample cleared with the OC method on a millimetre paper. (D) Fluorescence intensity vs. image depth (full field vs. region of interest—ROI). (E) 3D‐display of image data up to 1.6 mm depth of an YFP‐H‐line mouse brain, cleared with dithioglycerol at pH value of 8, and display of slices (maximum‐projection of throughout 30 µm) at different depth. The data shows that the here described PRIAMOS clearing procedure preserves the YFP‐fluorescence and makes the sample well transparent, enabling optical imaging down to 1.6 mm though the brain. The images were brightness‐adjusted to compensate for the decline in the signal strength at deeper positions. For better visualisation and noise reduction, a mild deconvolution was applied. A 3D animated version is shown in Video S1, online material (scale bars are 50 µm).

While dithioglycerol is only poorly soluble in water, monothioglycerol is miscible with water to some extent. Therefore, we still need an initial dehydration step for replacing the water from the sample with an organic solvent, for example, ethanol,^5^ tetrahydrofuran^15^, ^18^ or other higher alcohols^16^ prior to embedding in the procedure. First, we aimed to image a mouse brain (Figure 3). A schematic representation of the sample preparation process is provided in Figure 3A. A first test revealed that a clearing protocol with a dehydration (using tetrahydrofuran for dehydration) and clearing by immersing it in a mixture mono‐ and dithioglycerol with a refractive index of 1.56 resulted in samples with a good transparency (Figure 3B and C). However, the YFP‐fluorescence was not detectable anymore. Searching for a plausible explanation, we attributed the lack of YFP fluorescence to an unfavourable acid balance in the embedding medium: It is well‐known that YFP fluorescence is dependent on the sample pH value. Thiols, which are characterised by weak acidic properties, could therefore quench the fluorescence of the proteins. In order to assess the acidic properties, we measured the pH values of monothioglycerol and dithioglycerol in a 0.5% solution of the compounds in water. Measured values between 5 to 6 (500 µL of monothioglycerol in 100 mL dist., degassed water resulted in a pH value of 6.2, 500 µL dithioglycerol in 100 mL water featured a pH value of even 5.5) revealed a weak acidity, in which fluorescent proteins are not functional. We thus repeated this experiment and adjusted the pH values of all involved liquids to the range of 7 to 8 using a phosphate buffer in the first dehydration steps with a 50% and 70% v/v THF in water, and the base trietylamine for higher concentrations, with tributylamine for pure thioglycerols in order to compensate the acidic behaviour of thiols (Figure 2B). With the appropriate acidity, fluorescence was preserved, and we generated the same transparency as the first experiment.

We placed an isolated mouse brain of an YFP‐transgenic mouse (H‐line), cleared with the procedure described above, in an upright confocal microscope equipped with a BABB (*n* = 1.56) objective (Figure 3A). We selected confocal microscopy, being more predictable than light sheet microscopy, since there are less variations in the modalities (e.g., cylinder lens vs. scanned beam vs. dithering vs. single‐sided or double‐sided) and the penetration depth is only affected by the material above the sample and not the lateral extent of the sample as it would be in light‐sheet microscopy. The images are of a good quality even at 1 mm depth of the sample. This indicates that the mixture of the embedding medium matches well with the (*n* = 1.56) optical design conditions of the BABB objective lens. We did not observe excessive bleaching after acquiring a confocal image stack of 1200 images throughout 1.6 mm depth, which took about 6 h (Figure 3E). Figure 3D depicts a normalised intensity plot along the z‐direction of the CLSM dataset.

**FIGURE 4:**
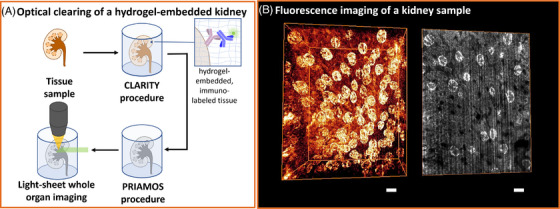
3D rendering of a rat kidney sample, cleared using the CLARITY‐protocol, and using pure monothioglycerol as index matched clearing substance. (A) Sample preparation of the kidney sample cut into a cube. For CLARITY, the sample is immuno‐labelled, and afterwards embedded into a hydrogel matrix. A treatment of the sample with surfactants enabled the removal of lipids, rendering the sample transparent. The PRIAMOS procedure homogenises the RI distribution within the sample further and enhances its overall RI, enabling whole organ imaging with a standard objective. (B) The slice on the right side visualises an image of the 3D stack at 600 µm depth (images size: 800 × 600 µm). The sample was stained for WT‐1, a protein expressed in the glomeruli of the kidney. The stripes are a typical artefact of light‐sheet microscopy.^36^ Scale bar: 100 µm.

We also applied PRIAMOS on kidney samples cleared with the CLARITY‐protocol and stained using immunochemistry (Figure 4). Kidney samples were cleared according to Unnersjö‐Jess et al.^35^ and stained for WT‐1 (Wilms’ tumour 1) using a rabbit polyclonal primary antibody (Santa Cruz, sc‐192, 1:200, 2 days incubation) and a goat anti‐Rabbit Alexa488‐conjugated secondary antibody (1:200, 2 days incubation). We imaged the sample with a light sheet microscope (Figure 4A), which required an embedding medium with a refractive index of *n* = 1.52, which is equal to pure monothioglycerol (Figure 2C). We transferred the CLARITY‐treated sample from the aqueous storage medium to the high‐RI medium thioglycerol by simply dropping it into pure monothioglycerol and obtained good results after some time (hours to 1 day as described in the experimental section) to allow for the diffusion of monothioglycerol into the sample (Figure 4A). An adjustment of the pH value was not necessary in this case, as the samples were labelled with Alexa488‐conjugated antibodies, whose fluorescence is not sensitive to decreased pH levels. A detailed analysis of the transparency at a larger depth was not possible with this imaging apparatus, as the objective lens had a limited working distance of 1 mm. However, the data shows that monothioglycerol with a refractive index of close to immersion oil (*n* = 1.52) is a good alternative for the literature‐known CUBIC‐R.^44^

## 4 DISCUSSION

We increased the refractive index of glycerol, a substance already used for sample embedding, by applying a substance which we have identified using the PRIAMOS method (Figure 1). Briefly, the hydroxyl‐groups of glycerol are substituted by thiol‐groups (Figure 2). As monothioglycerol and dithioglycerol exhibit an RI of *n* = 1.52 and 1.574 and both substances are miscible with each other at all ratios, refractive indices between these two values can be freely adjusted following a linear trend (Figure 2C). We can additionally add tributylamine to allow for adjusting the acidity of the mixture to mimic an environment with a neutral to basic pH value, where fluorescent proteins are functional. Adding tributylamine with a refractive index of *n* = 1.43 is a trade‐off between a high refractive index and optimal acidic balance regarding the fluorescence performance. Our mixture with a refractive index of BABB (*n* = 1.56) resulted in directly measured pH value of 8, and of 7.1 in the 0.5% aqueous dilution, which is sufficiently basic to make the fluorescent protein YFP functional. Note that we needed to adjust the acidity to pH values of 7 to 9 to maintain the fluorescence of the proteins GFP^45^ and YFP. We then imaged the sample using a dedicated objective lens for BABB‐immersion (Figure 3). The sample is highly transparent and fluorescent proteins remained functional, and imaging down to a depth of 1.6 mm in the murine brain was possible (Figure 3E). As indicated in a fluorescence intensity plot, the fluorescence intensity was retained to approx. 25% of its maximum at an imaging depth of approx. 1.5 mm and is reduced to an acceptable intensity at 1.6 mm.

We also used pure monothioglycerol as embedding medium for a sample cleared with the CLARITY‐protocol and imaged it with a light‐sheet microscope with illumination optics for embedding media with a refractive index of 1.52. This sample treatment also resulted in a good transparency, and imaging down to 0.6 mm remained unproblematic. We are confident, that even larger imaging depths can be achieved using suitable objective lenses. In order to roughly estimate the acidity of the embedding medium, we measured an aqueous solution of the refractive index liquid, confirming that the medium diluted in water buffers the solution to neutral pH conditions (pH value of 7.1). Our acidity considerations proved to be right since YFP became functional when we made the embedding medium more basic with the help of tributylamine. Note that the pH scale from 0 to 14 is defined in an aqueous environment.

For a highly precise adjustment of the refractive index by mixing two substances for a specific wavelength, we would also have to consider chromatic dispersion. The chromatic dispersion describes the change of refractive index with the wavelength of light. This is modelled by the Abbe‐number, which represents an important parameter in optical design. The immersion medium BABB is of a low Abbe number between 25 and 28 (the Abbe values of benzylbenzoate and benzylalcohol, calculated by the formula for the Abbe‐number, and using the refractive index database^46^). Dithioglycerol and monothioglycerol possess Abbe numbers of 36 of 43,^46^ while Abbe numbers for glasses are between 25 and 80. Although the Abbe values of BABB and dithioglycerol are similar on this scale, the small difference would have to be considered when we want to match the refractive index of a mixture of liquids precisely to the refractive index of BABB at a specific wavelength.

However, when we purposely matched the refractive index of the mixture of dithioglycerol and tributylamine to *n* = 1.563 instead of *n* = 1.559, the refractive index that was used in the design of the dedicated BABB objective lens, the confocal measurement still provided good results. We assume this is because the detection pinhole in the confocal measurement optics corrects for aberrations caused by refractive index mismatch, rendering the measurement insensitive to small Abbe number mismatches.

We observed a modest shrinking of the sample. This could be caused by dissolving lipids and removing them from the sample in the dehydration step, where we replace the water with the solvent THF, being a good solvent for lipids. However, we deem the shrinkage in a moderate and acceptable range. Other techniques are optimised for preserving the sample size, as this is needed for the subsequent image registration.^22^ We do not apply such image registration techniques, and therefore we also do not need to conserve the volume exactly.

This clearing protocol is not compatible with the ‘CLARITY’‐protocol,^13^ in that sense that the hydrogel‐embedded sample is subjected to dehydration with THF and subsequently immersed in mono‐ and dithioglycerol. During the dehydration step, the sample collapses and the sample becomes intransparent. The ‘CLARITY’‐embedded sample, however, could be embedded in the thioglycerols directly. During the immersion procedure, the thioglycerols did not affect the acrylamide‐polymer used in the ‘CLARITY’‐technique.

Thioglycerols display a higher chemical reactivity than other organic substances used clearing agents, for example, the inert substances BABB^5^ or dibenzylether^18^ or the water‐soluble substances.^6^, ^7^, ^8^, ^9^, ^10^, ^22^ According to the delivery note these substances should be stored in the fridge. Dithioglycerol and tributylamin are toxic and have an uncomfortable smell, but they are not very volatile with boiling points at 140 and 210°C, which simplifies the handling of these substances. However, the toxicity of the substances requires handling of the embedding in a suction drainage close to the sample chamber of the microscope, and to accomplish the clearing protocol in a fume hood. The reactive properties of thioglycerols, however, also have a pivotal advantage: The redox‐activity of sulphur can be used as a scavenger of reactive oxygen species. As these may emerge from photochemical activation of a sample, they are largely responsible for photobleaching. Thiol derivatives have the ability to eliminate the reactive species, rendering them efficient anti‐fading substances.^47^

## 5 CONCLUSIONS

In summary, we devised the PRIAMOS‐technique to find suitable substances for clearing biological samples. The PRIAMOS principle is based on replacing atoms in well‐known embedding media with atoms that possess larger, thus, more easily polarisable orbitals of a molecule without changing its molecular structure, and therefore mimicking an environment where the fluorescence protein functionality was previously reported. Accordingly, we exchanged hydroxy functions from the well‐known embedding medium glycerol by thiols, thus, using mono‐ and dithioglycerol, enabling a free adjustment of the RI between 1.52 and 1.574. This first mixture of substances devised from this principle showed good results in terms of transparency, and after adjusting the pH value, the functionality of genetic fluorescence was maintained. As this worked even with the first liquid substance we tried, other substances can be devised that might yield further improvements. The PRIAMOS principle provides a good guideline in what direction the search for suited substances for index‐matching should be directed.

## CONFLICT OF INTEREST STATEMENT

The authors declare no conflicts of interest.

## Supporting information



Supporting information
